# Prognostic Values of Transforming Growth Factor-Beta Subtypes in Ovarian Cancer

**DOI:** 10.1155/2020/2170606

**Published:** 2020-04-12

**Authors:** Junhan Zhou, Wenxiao Jiang, Wenbin Huang, Miaomiao Ye, Xueqiong Zhu

**Affiliations:** Department of Obstetrics and Gynecology, The Second Affiliated Hospital of Wenzhou Medical University, Wenzhou, Zhejiang 325027, China

## Abstract

**Purpose:**

To explore the potential role of the transforming growth factor-beta (TGF-*β*) subtypes in the prognosis of ovarian cancer patients. *Materials and Methods*. The prognostic roles of individual TGF-*β* subtypes in women with ovarian cancer were retrieved from the Kaplan-Meier plotter (KM plotter) database. In addition, the Oncomine database and immunohistochemistry were used to observe the mRNA and protein expression of TGF-*β* subtypes between human ovarian carcinoma and normal ovarian samples, respectively.

**Results:**

TGF-*β*1 and TGF-*β*4 were totally uncorrelated with survival outcomes in women with ovarian cancer. Increased TGF-*β*2 and TGF-*β*3 mRNA expression was markedly related to unfavorable prognosis, especially in women with serous, poorly differentiated, and late-stage ovarian carcinoma. High expression levels of TGF-*β*2 were related to worse progression-free survival (PFS) while TGF-*β*3 was linked to unfavorable overall survival (OS) and PFS in women with TP53-mutated ovarian cancer. TGF-*β*2 was associated with poor OS and PFS from treatment with chemotherapy with platins, Taxol, or a platin+Taxol. However, overexpression of TGF-*β*3 was associated with poor OS from the use of platins and poor PFS of Taxol or a platin+Taxol in women with ovarian carcinoma. Furthermore, the expression of TGF-*β*2 mRNA and protein was higher but only TGF-*β*3 mRNA expression was higher in cancerous tissues than in normal ovarian samples.

**Conclusion:**

Higher expression of TGF-*β*2 functioned as a significant predictor of poor prognosis in women with ovarian cancer, especially those with TP53 mutations or who were undergoing chemotherapy with platins, Taxol, or a platin+Taxol.

## 1. Introduction

Ovarian cancer is one of the three major gynecological malignancies and ranks the highest in mortality worldwide, with 22,240 new cases and approximately 140,700 cancer-related deaths annually [1]. The occurrence of the disease is concealed owing to the absence of an effective screening method in early stages, resulting in extensive metastasis when discovered. Although a comprehensive treatment based on debulking surgery is applied to advanced ovarian cancer in combination with postoperative chemotherapy, the five-year survival rate remains merely 30% [2]. The identification of potential prognostic biomarkers and innovative therapeutic targets of ovarian cancer is urgently needed to enhance the clinical outcomes for women with ovarian cancer.

Human transforming growth factor-beta (TGF-*β*) consists of four identified subtypes, including TGF-*β*1, TGF-*β*2, TGF-*β*3, and TGF-*β*4. TGF-*β*, which is a pleiotropic cytokine with complex functions, promotes the transformation of fibroblasts and regulates many significant biological behaviors including cell proliferation, apoptosis, metastasis, and differentiation [3]. TGF-*β* binding to its receptor initiates intracellular activation via the phosphorylated transcription factor Smad. TGF-*β* also activates other intracellular signal transduction pathways, including pathways related to mitogen-activated protein kinases (MAPKs), phosphatidylinositol 3-kinase (PI3K), and Ras superfamily small GTPases, to indirectly regulate the Smad pathway [4, 5].

TGF-*β* has been identified to be overexpressed in a variety of cancer tissues and cancer cell lines and has also been well acknowledged as a prognostic predictor for various carcinomas, such as hepatocellular carcinoma [6], glioma [7], colorectal carcinoma [8], and oral squamous cell carcinoma [9]. For example, the expression of TGF-*β*1 was shown to be related to worse differentiation and shorter median survival time in glioma carcinoma [7]. It was well known that TGF-*β*2 acts as an inhibitor in early stages and a promoter in advanced stages of breast cancer, and TGF-*β*2mRNA was negatively correlated with its protein expression [10]. It was reported that ovarian cancer cells could lose their response to the inhibitory functions of TGF-*β* and promote the cell metastasis and epithelial-mesenchymal transition (EMT) [11]. However, researches about TGF-*β* as the predicted markers related to prognosis of TGF-*β* in ovarian cancer are limited. Hence, this study was designed to explore the prognostic value of four subtypes of TGF-*β* in women with ovarian cancer.

## 2. Materials and Methods

### 2.1. Kaplan-Meier (KM) Plotter Database

The online Kaplan-Meier (KM) plotter database (http://kmplot.com/analysis/) contains gene expression and clinical data, and this database currently contains the survival information of a total of 54,675 genes in the use of 10,461 carcinoma specimens with a mean follow-up of 40 months currently. Gene expression data and overall survival (OS) and progression-free survival (PFS) information were downloaded from the Gene Expression Omnibus (GEO), the European Genome-phenome Archive (EGA), and The Cancer Genome Atlas (TCGA). OS was defined as the time from randomization to death for any reason. PFS referred to the length of time between the patients entering the trials and the tumor progressing or patients death. The online databases were used to evaluate the relationship between TGF-*β* mRNA expression and OS and PFS in women with ovarian cancer.

From analyzing the prognostic significance of individual TGF-*β* subtypes (TGF-*β*1, TGF-*β*2, TGF-*β*3, and TGF-*β*4) in ovarian cancer, four subtypes of TGF-*β* were entered into the database in turn. The patients were subgrouped as “low” and “high” on the basis of the mRNA expression values with established cutoffs for ovarian carcinoma samples [12]. KM survival plotter was used to test the difference between two cohorts of patients. The hazard ratios (HRs), 95% confidence intervals (CIs), and *P* values were estimated. A *P* value < 0.05 was considered significant. The OS and PFS information for ovarian cancers in terms of grade, stage, histology, TP53 mutation status, and debulking and chemotherapy strategies were further studied in our research.

### 2.2. Oncomine Database

To further clarify the mRNA expression level of TGF-*β* subtypes in ovarian cancer, our study used the Oncomine database (https://www.oncomine.org) for analysis. The Oncomine database is a publicly accessible and universally searchable online data-mining platform with carcinoma microarray expression data from whole-genome oligonucleotide array differential expression analysis [13, 14]. The search parameters we input were as follows: analysis type (ovarian cancer vs. ovarian normal tissue), cancer type (ovarian cancer), data type (mRNA), and gene. The other parameters were set as systematic defaults. Eight cases of normal ovarian epithelial tissues and 586 ovarian serous cyst adenocarcinoma samples were used. We compared the different mRNA expression of TGF-*β* subtypes in normal tissue and cancer tissue and used the cutoff threshold of a *P* value < 0.05, fold changes ≥ 2-fold, and gene rank in the top 10% to identify the top genes, and the results were shown in the form of a box plot.

### 2.3. Immunohistochemistry

Immunohistochemistry was carried out on the tissue sections (4 *μ*m) from 10 formalin-fixed, paraffin-embedded serous ovarian cancer tissues and 10 normal ovarian tissues, which were all pathologically confirmed. After deparaffinization in xylene and rehydration in an alcohol series, the slides were subjected to antigen retrieval, incubated in 0.3% hydrogen peroxide in methanol, and then blocked with normal goat serum (10%). Next, the sections were incubated with primary antibodies against TGF-*β*2 protein (diluted 1 : 1000, Abcam, Cambridge, UK) and anti-TGF-*β*3 protein (diluted 1 : 1000, Abcam, Cambridge, UK) at 4°C overnight. After rinsing in PBS for three times, the slides were incubated with biotinylated goat anti-mouse antibody and subsequently detected with 3′3-diaminobenzidine tetrahydrochloride (DAB) (1 : 50 dilution, GIBCO) staining and counterstained with hematoxylin. Positive and negative controls were set for each experiment.

The staining intensity (SI) was observed and scored by two pathologists blindly. The staining extent was recorded as a score of 0 with 0%positively stained cells, a score of 1 with 1%–25% stained cells, a score of 2 with 26%–50% stained cells, a score of 3 with 51%–75% stained cells, and a score of 4 with 76%–100% stained cells. The intensity of positive staining was graded as 0 with no staining, 1 with light yellow staining, 2 with yellow staining, and 3 with brown staining. Finally, the protein expression of TGF-*β*2 and TGF-*β*3 was evaluated by multiplying the percentage of positively stained cells by the staining intensity (score ranged from 0 to 12). The average value from the two referees was used as the final score.

### 2.4. Statistical Analysis

Statistical analyses were performed with SPSS 17.0 software (SPSS, Chicago, USA). Data was expressed as the mean ± standard deviation of the mean (SD), and Student's *t*-test was used for group comparisons. *P* < 0.05 was considered statistically significant.

## 3. Results

### 3.1. The Expression of TGF-*β*1 and TGF-*β*4 Was Unrelated to Survival in All Ovarian Cancer Patients

**Figure 1:**
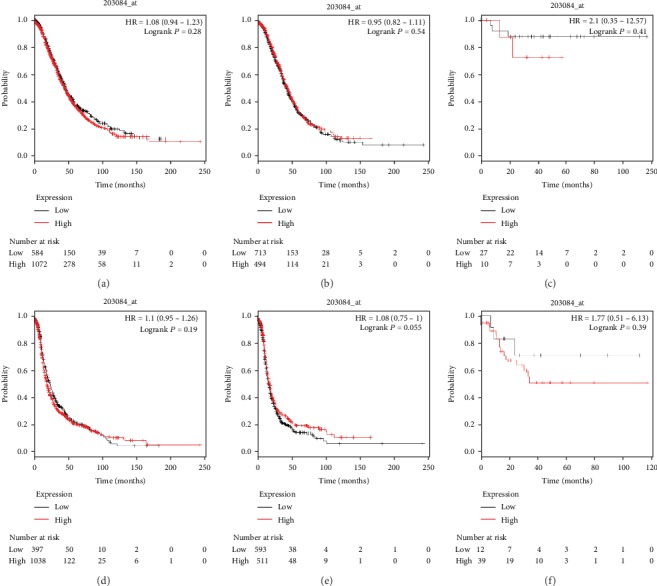
The prognostic value of TGF-*β*1 for predicting OS and PFS in ovarian cancer patients. OS curves were plotted for all ovarian cancer patients (*N* = 1,656, a), serous ovarian cancer patients (*N* = 1,207, b), and endometrioid ovarian cancer patients (*N* = 37, c); PFS curves were plotted for all ovarian cancer patients (N = 1,435, d), serous ovarian cancer patients (*N* = 1,104, e), and endometrioid ovarian cancer patients (*N* = 51, f).

The prognostic value of TGF-*β*1 was initially explored in the database (Affymetrix ID: 203084_at). As shown in Figure 1, increased TGF-*β*1 mRNA expression had no relationship with OS according to the OS curves for TGF-*β*1 and also had no association with PFS according to the PFS curves for all women with ovarian carcinoma, endometrioid ovarian cancer, and serous ovarian cancer (all *P* > 0.05).

**Figure 2:**
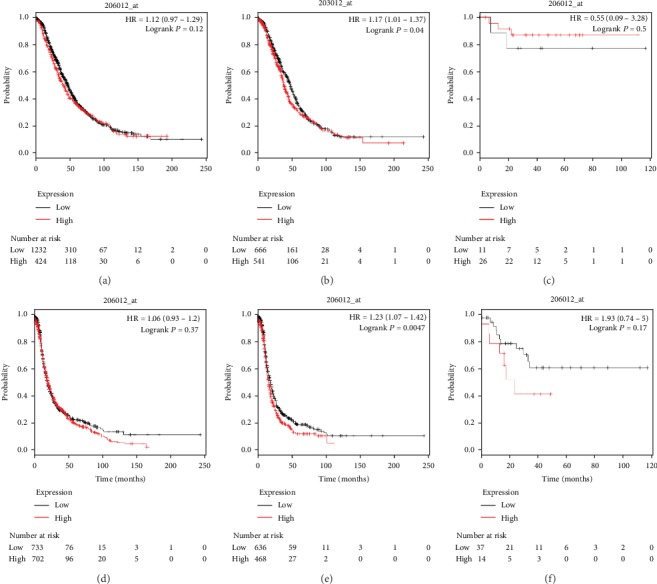
The prognostic value of TGF-*β*4 for predicting OS and PFS in ovarian cancer patients. OS curves were plotted for all ovarian cancer patients (*N* = 1,656, a), serous ovarian cancer patients (*N* = 1,207, b), and endometrioid ovarian cancer patients (*N* = 37, c); PFS curves were plotted for all ovarian cancer patients (*N* = 1,435, d), serous ovarian cancer patients (*N* = 1,104, e), and endometrioid ovarian cancer patients (*N* = 51, f).

The prognostic value of TGF-*β*4 (Affymetrix ID: 206012_at) was demonstrated in OS and PFS curves for TGF-*β*4. As shown in Figure 2, elevated TGF-*β*4 mRNA expression was related to poor OS and PFS in women with serous ovarian cancer (HR, 1.17; 95% CI, 1.01–1.37; *P* = 0.04) (HR, 1.23; 95% CI, 1.07–1.42; *P* = 0.0047) but exhibited no significant relationship with OS or PFS in women with all ovarian carcinoma and women with endometrioid ovarian carcinoma.

### 3.2. Elevated mRNA Levels of TGF-*β*2 and TGF-*β*3 Were Related to Poor OS and PFS in Ovarian Cancer Patients

**Figure 3:**
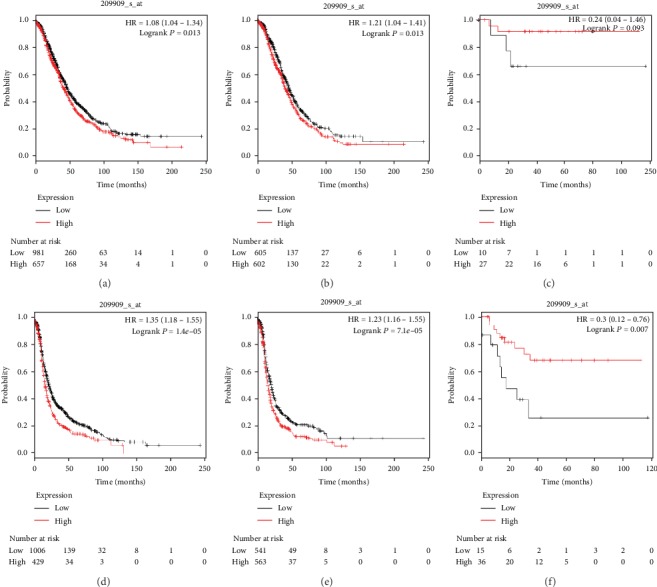
The prognostic value of TGF-*β*2 for predicting OS and PFS in ovarian cancer patients. OS curves were plotted for all ovarian cancer patients (*N* = 1,656, a), serous ovarian cancer patients (*N* = 1,207, b), and endometrioid ovarian cancer patients (*N* = 37, c); PFS curves were plotted for all ovarian cancer patients (*N* = 1,435, d), serous ovarian cancer patients (*N* = 1,104, e), and endometrioid ovarian cancer patients (*N* = 51, f).

The prognostic significance of TGF-*β*2 was assessed (Figure 3) using the corresponding Affymetrix ID: 209909_s_atdatase. Elevated levels of TGF-*β*2 expression were related to unfavorable OS and PFS for all women with ovarian carcinoma (HR, 1.18; 95% CI, 1.04–1.34; *P* = 0.013; and HR, 1.35; 95% CI, 1.18–1.55; *P* = 0.001, respectively), as well as for women with serous ovarian carcinoma (HR, 1.21; 95% CI, 1.04–1.41; *P* = 0.013; and HR, 1.34; 95% CI, 1.16–1.55; *P* = 0.001, respectively). In addition, TGF-*β*2 mRNA overexpression showed worse PFS than lower levels of TGF-*β*2 mRNA expression in women with endometrioid ovarian carcinoma (HR, 0.30; 95% CI, 0.12–0.76; *P* = 0.007) but showed no difference in OS in women with endometrioid ovarian carcinoma women (HR, 0.24; 95% CI, 0.04–1.46; *P* = 0.093).

**Figure 4:**
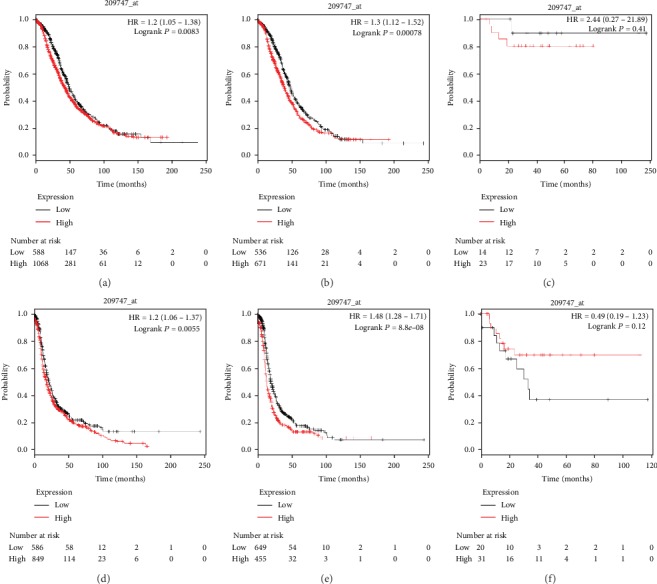
The prognostic value of TGF-*β*3 for predicting OS and PFS in ovarian cancer patients. OS curves were plotted for all ovarian cancer patients (*N* = 1,656, a), serous ovarian cancer patients (*N* = 1,207, b), and endometrioid ovarian cancer patients (*N* = 37, c); PFS curves were plotted for all ovarian cancer patients (*N* = 1,435, d), serous ovarian cancer patients (*N* = 1,104, e), and endometrioid ovarian cancer patients (*N* = 51, f).

As presented in Figure 4, the prognostic value of TGF-*β*3 (Affymetrix ID: 209747_at) was investigated. Elevated TGF-*β*3 mRNA expression predicted poor OS and PFS in all women with ovarian cancer (HR, 1.20; 95% CI, 1.05–1.38; *P* = 0.0083; and HR, 1.20; 95% CI, 1.06–1.37; *P* = 0.0055, respectively) and in women with serous ovarian cancer (HR, 1.30; 95% CI, 1.12–1.52; *P* = 0.0008; and HR, 1.48; 95% CI, 1.28–1.71; *P* = 0.001, respectively). Nevertheless, with regard to women with endometrioid ovarian cancer, TGF-*β*3 mRNA expression had no significant effect on OS (HR, 2.44; 95% CI, 0.27–21.89; *P* = 0.41) or on PFS (HR, 0.49; 95% CI, 0.19–1.23; *P* = 0.12).

### 3.3. Prognostic Significance of TGF-*β*2 and TGF-*β*3 mRNA Expression in Ovarian Tumors with Other Different Clinicopathologic Features

As shown in Table 1, increased TGF-*β*2 mRNA expression was related to a favorable OS but not associated with PFS in women with stage I and II ovarian cancer, while upregulated TGF-*β*2 mRNA levels were related to poor OS and PFS in women with stage III and IV ovarian carcinoma. Moreover, upregulated TGF-*β*3 expression was significantly associated with poor PFS in stage III and IV ovarian cancer patients while it showed no association with OS in stage III or IV ovarian cancer patients.

Table 2 shows that increased TGF-*β*2 expression was related to poor OS and PFS in women with grade III ovarian carcinoma and to negative OS in women with grade II ovarian carcinoma. High expression of TGF-*β*3 mRNA was related to poor OS and PFS in women with grade II ovarian carcinoma and correlated with poor PFS in women with grade III ovarian carcinoma.

Regarding TP53 mutation status, TGF-*β*2 only predicted a poor PFS in women with TP53-mutated ovarian carcinoma. Increased expression of TGF-*β*3 predicted unfavorable OS and PFS in women with TP53-mutated ovarian cancer as shown in Table 3.

For the chemotherapy strategies shown in Table 4, elevated TGF-*β*2 mRNA expression was linked to poor OS and PFS in women with ovarian carcinoma treated with platins, Taxol, or a platin+Taxol. However, upregulated TGF-*β*3 expression was correlated with a favorable PFS but a poor OS in women with ovarian carcinoma undergoing chemotherapy with platins. Among women with ovarian cancer treated with Taxol or a platin+Taxol, overexpression of TGF-*β*3 was related to poor PFS.

### 3.4. Different mRNA Expression Levels of TGF-*β* Subtypes in Ovarian Cancer and Normal Ovarian Tissues

**Figure 5:**
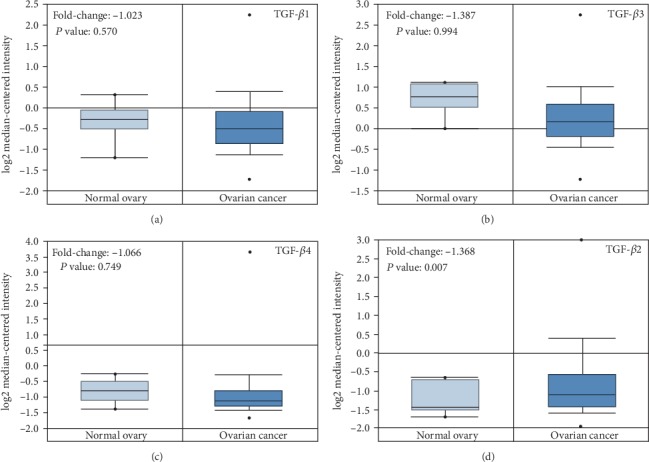
mRNA expression levels of TGF-*β* subtypes in ovarian cancer and normal ovarian tissues. (a) TGF-*β*1, (b) TGF-*β*3, (c) TGF-*β*4, and (d) TGF-*β*2.

As was shown in the box plot in the Oncomine database, the TGF-*β*1, TGF-*β*3, and TGF-*β*4 mRNA expression levels between ovarian carcinoma and normal ovarian tissues showed no significant differences (*P* = 0.57, Figure 5(a); *P* = 0.994, Figure 5(b); and *P* = 0.749, Figure 5(c), respectively). Notably, TGF-*β*2 presented a higher mRNA transcription level (fold change of 1.368) in cancerous tissues than in normal tissues (*P* = 0.007; Figure 5(d)).

### 3.5. The Protein Levels of TGF-*β*2 and TGF-*β*3 in Human Ovarian Cancer and Normal Ovarian Tissues

**Figure 6:**
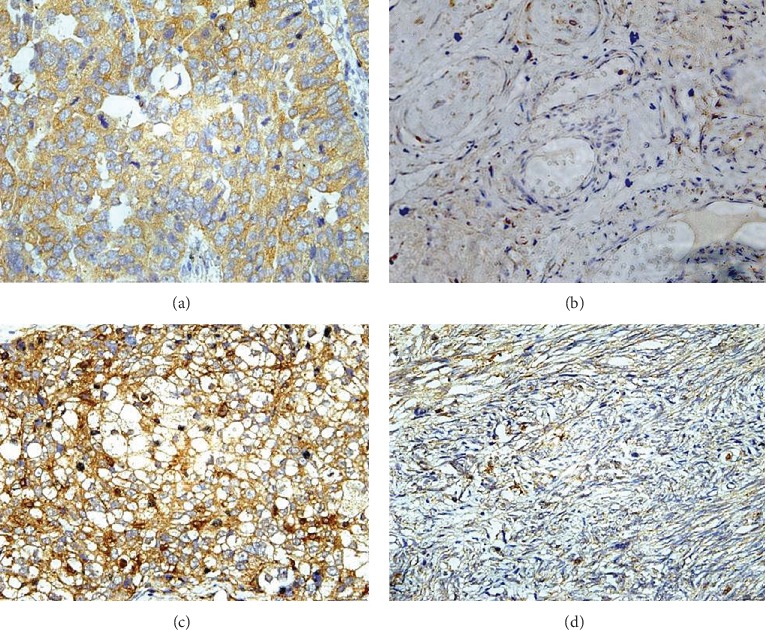
The protein expression of TGF-*β*2 and TGF-*β*3 in human ovarian cancer and normal ovarian tissues. TGF-*β*2 protein expression in ovarian cancer tissues (a) and in normal ovarian tissues (b).TGF-*β*3 protein expression in ovarian cancer tissues (c) and in normal ovarian tissues (d).SP staining, ×4.

Based on the differential mRNA expression of TGF-*β*2 between cancer and normal tissues, as well as the significant correlation of TGF-*β*2 and TGF-*β*3with survival, the expression of the different proteins was assessed. As shown in Figure 6, TGF-*β*2 and TGF-*β*3 staining was seen in the cytoplasm of positive cells, in both the ovarian carcinoma and normal ovarian samples. Additionally, the expression of TGF-*β*2 staining score in ovarian cancer tissues (8.53 ± 1.24) was relatively higher than that in normal ovarian tissues (3.03 ± 1.34) (*P* < 0.001; Figures 6(a) and 6(b)). To some extent, the TGF-*β*3 protein expression in ovarian cancer tissues (7.64 ± 0.74) was also significantly greater than that in normal ovarian samples (3.92 ± 0.38) (*P* < 0.001; Figures 6(c) and 6(d)).

## 4. Discussion

To confirm the prognostic significance of TGF-*β* subtypes in women with ovarian tumors, our research explored the correlation between the expression level of TGF-*β* subtypes and the survival of ovarian carcinoma patients. It was found that TGF-*β*2 and TGF-*β*3 mRNA levels were related to poor prognostic outcomes, while TGF-*β*1 and TGF-*β*4 had no association with prognosis in women with ovarian carcinoma. Hence, the relationships between TGF-*β*2 and TGF-*β*3 and different clinicopathologic features of ovarian cancer were comprehensively assessed. In addition, immunohistochemistry results confirmed a significantly higher expression of TGF-*β*2 in ovarian carcinoma samples than in normal ovarian samples.

TGF-*β*1 is a powerful immunosuppressant in humans that inhibits cell growth and is an anti-inflammatory during the early stages of carcinomas [15]. Abnormal activation of TGF-*β*1 in the late stages of gastric cancer has been revealed to promote the development of aggressive growth and metastases of primary gastric carcinomas by regulating paracrine effects on mesenchymal cells, vascular endothelial cells, and lymphocytes [16]. A higher level of TGF-*β*1 expression predicted worse clinical outcomes in hepatitis B virus-related (HBV) hepatocellular carcinoma patients [6]. Regarding the role of TGF-*β*1 in ovarian cancer, Yan et al. [17] revealed that TGF-*β*1 was increased in epithelial ovarian cancer (EOC) tissues and was positively related to poor differentiation grades and advanced FIGO stages. However, Tas et al. [18] observed no significant difference between EOC patients and healthy persons in TGF-*β*1 expression level. Meanwhile, TGF-*β*1 was found to have no relationship with OS and PFS in women with EOC. Wang et al. [19] observed that azoxymethane induced colon tumors in mice through the alterations of TGF-*β*1 and its type II receptor (TbetaR-II). In contrast to the levels in the control mouse normal colon tissues, the mRNA expression levels of TGF-*β*1 and TbetaR-II increased 1.8-fold and 1.3-fold, respectively, in the mice with tumors. Meanwhile, the immunohistochemistry results showed an increase in the staining intensity of both TGF-*β*1 and TbetaR-II in colon cancer tissues, which correlated with the mRNA expression level. Similar to the results of Taset al., our study observed that TGF-*β*1 had no association with the prognosis of ovarian cancer patients, but further study of TGF-*β*1 in ovarian cancer subtypes is required.

TGF-*β*4, also called endometrial bleeding-associated factor, is an important gene of the TGF-*β* superfamily. It showed high expression specifically in the endometrium in women in the late secretory phase, and weak expression was also observed in the colon, duodenum, ovary, and pancreas [20]. Meanwhile, seminomas and embryonal tumors in the testis and adenocarcinomas of the ovary and colon all showed high expression of TGF-*β*4 [20]. To date, the specific studies about the relationship between TGF-*β*4 and prognosis in cancers are limited. There have been no reports about the relationship between TGF-*β*4 expression and the prognosis of ovarian cancer. Our study demonstrated that the expression of TGF-*β*4 had no relationship with the clinical outcomes of ovarian carcinoma patients. This suggests that more research on prognostic significance of TGF-*β*4 in various cancers, especially in ovarian tumors, is needed.

A variety of papers have observed the relationship between TGF-*β*2 expression and carcinomas in recent years, showing that increased TGF-*β*2 expression predicts a worse prognosis in breast cancer [10], gastric carcinoma [21], non-small-cell lung cancer [22], prostate cancer [23], and glioblastoma [24]. Vagenas et al. [25] studied 110 gastric cancer tissues and found that TGF-*β*2 was highly expressed in late stages and linked to poor prognosis, and upregulated expression of TGF-*β*2 promoted the progression of gastric tumors. Mechanistically, Do et al. [26] found that TGF-*β*2 induced the secretion of matrix metalloproteinase (MMP), loss of cell junctions, upregulation of N-cadherin, and downregulation of E-cadherin to enhance the potential of ovarian carcinoma cell metastasis. In line with this finding, Bilandzic et al. [27] demonstrated that ovarian granulosa cell tumors had high expression of MMP2 which depended on the stimulation of TGF-*β*2 in a nuclear factor-*κ*B (NF-*κ*B-) dependent manner. The NF-*κ*B/TGF-*β*2 signaling pathway contributed to the metastasis of granulosa cell tumors in the early stages. It is speculated that TGF-*β*2 may be an important predictive tumor marker. To date, studies about the prognostic values of TGF-*β*2 and the differential expression between normal tissues and cancerous tissues in ovarian cancer have not been performed. Our research showed that high mRNA expression of TGF-*β*2 was related to poor outcomes in women with ovarian carcinoma, particularly in women with grade III, stage III and IV, serous ovarian carcinoma. Furthermore, the expression of TGF-*β*2 in ovarian carcinoma samples was higher than that in normal ovarian samples at both mRNA and protein levels, which was different from the research by Dave et al. [10]. They found that TGF-*β*2 mRNA levels, with higher expression seen in advanced breast cancer tumors than in early-stage cancer, were inversely related to TGF-*β*2 protein levels, although TGF-*β*2 mRNA and protein levels were both related to clinicopathologic prognosticators. Therefore, TGF-*β*2 may be a significant biomarker of poor prognosis in women with ovarian carcinoma, especially for advanced stage, poorly differentiated, and serous ovarian cancer patients. The consistent expression of TGF-*β*2mRNA and protein also suggests that TGF-*β*2 plays a vital role in the poor prognosis of ovarian tumors at the transcript level. However, the signaling pathway of abnormal TGF-*β*2 expression in ovarian tumors is still unclear and needs further research.

TGF-*β*3 has a conserved sequence construction, and the mRNA expression of TGF-*β*3 is mainly derived from mesenchymal cells [28]. Studies on the prognostic value of TGF-*β*3 in malignant tumors are rare. Ghella et al. [29] studied 153 invasive breast cancer samples and found that upregulated TGF-*β*3 expression was inversely related to OS in patients with breast cancer, especially in patients with node metastases, suggesting that TGF-*β3* may be used for predicting poor prognosis in breast cancer patients. In the present study, we investigated whether TGF-*β*3 was correlated with poor outcomes in all women with ovarian carcinoma and particularly in women with serous ovarian carcinoma. Furthermore, we found that high TGF-*β*3 mRNA levels predicted a decreased survival rate in women with grade II and III ovarian carcinoma, suggesting that TGF-*β*3 is worse at predicating prognosis in poorly differentiated and serous ovarian tumor patients. To date, a correlation between TGF-*β*3 expression at the mRNA and protein levels and the prognosis of carcinoma has not been found in the literature. In our research, TGF-*β*3 expression was detected at a higher level in ovarian carcinoma samples than in normal samples at the protein level, but there was no significant difference between tumor samples and normal ovarian samples at the mRNA level. The differential expression of numerous genes between cancerous and normal tissues may also be inconsistent at the mRNA level and at the protein level. Proteins, as the executors of function, are regulated at the posttranscription level, and their precise mechanisms or pathways remain unclear and need to be further studied. Differential protein expression between cancerous and normal tissues thus possesses meaningful significance. This suggests that TGF-*β*3 may be an important predictive marker for poor prognosis at the posttranscriptional level.

p53 is a tumor suppressor gene that has been studied extensively. Emerging evidence has demonstrated that p53 affects the processes of tumor invasion, metastasis, and epithelial-mesenchymal transition (EMT) by regulating the TGF-*β* signaling pathway. For example, Lam et al. [30] found that p53 suppressed TGF-*β*3-induced metastasis, invasion, and EMT in normal epithelial tissues and cancerous breast cells. However, research on the relationship between TGF-*β*2 and p53 is limited. In this study, it was found that overexpression of TGF-*β*3 indicated poor outcomes while a high expression level of TGF-*β*2 was correlated with unfavorable PFS in ovarian carcinoma patients with TP53 mutation, but not in patients with TP53-wild-type ovarian carcinoma, implying that TGF-*β*2 and TGF-*β*3 might be poor predictors in women with TP53-mutated ovarian carcinoma.

Tang et al. [31] reported that hypoxia-inducible factor-1*α* (HIF-1*α*) and TGF-*β*2 were secreted by cancer-associated fibroblasts which promoted the strong expression of glioma-associated oncogene protein-2 in cancer stem cells; this protein is related to the chemoresistance to the combination of 5-fluorouracil and oxaliplatin and relapse of colorectal cancer following chemotherapeutic strategies. Similarly, Bhola et al. [32] investigated how the TGF-*β* signaling pathway increased the relapse of breast carcinoma through IL-8-induced expansion of cancer stem cells and stimulation of the development of chemoresistant cancer stem cells to paclitaxel. Specifically in ovarian cancer, one study by Hong et al. [33] discovered that downregulation of liver kinase b1 (LKB1) stimulated TGF-*β* expression and EMT, which led to the resistance of chemotherapy of ovarian cancer cells to chemotherapy. Collectively, TGF-*β* might significantly increase chemoresistance in cancer chemotherapy treatment. In our current study, we found that elevated TGF-*β*2 expression was related to poor outcomes in women with ovarian carcinoma receiving chemotherapy with platins, Taxol, or a platin+Taxol. Overexpression of TGF-*β*3 was related to poor OS after treatment with platins and poor PFS after treatment with Taxol or a platin+Taxol in ovarian cancer patients. Overexpression of TGF-*β*2 and TGF-*β*3 in ovarian cancer tissues could predict poor prognosis in ovarian cancer patients treated with chemotherapy. Therefore, TGF-*β*2 and TGF-*β*3 are effective predictors of the efficacy of platins and platin-based anticancer therapeutics in ovarian cancer patients.

## 5. Conclusion

The expression of TGF-*β*1 and TGF-*β*4 had no association with the prognosis of women with ovarian cancer; nevertheless, high expression of TGF-*β*3 may be related to poor prognosis, and TGF-*β*3 may exert its functions at on the posttranscriptional level, but this mechanism needs more study in ovarian cancer. Overexpression of TGF-*β*2 functioned as a predictive biomarker of poor prognosis in ovarian cancer, especially for serous, poorly differentiated, and advanced-stage ovarian carcinomas. Meanwhile, increased TGF-*β*2 expression at both the mRNA and protein levels was found to be related to poor prognosis in women with TP53-mutated ovarian cancer as well as in cancer patients receiving chemotherapy with platins, Taxol, or a platin+Taxol. The discovery of inhibitors for TGF-*β*2 target gene inhibitors might be an efficient way to enhance the clinical outcomes of women with ovarian cancer.

## Figures and Tables

**Table 1:** Correlation of TGF-*β* subtypes with OS and PFS in ovarian cancer patients with different clinical stages of disease.

TGF-*β* subtypes	Clinical stages	Cases	OS	*P* value	Cases	PFS	*P* value
			HR (95% CI)			HR (95% CI)	
TGF-*β*1	I+II	135	2.62 (1.20-5.73)	0.0012^∗^	163	2.40 (1.36-4.23)	0.0018^∗^
	III+IV	1220	1.11 (0.94-1.30)	0.21	1081	0.85 (0.74-0.98)	0.023^∗^

TGF-*β*2	I+II	135	0.40 (0.18-0.98)	0.021^∗^	163	0.72 (0.41-1.28)	0.26
	III + IV	1220	1.21 (1.04-1.40)	0.011^∗^	1081	1.29 (1.12-1.48)	0.0004^∗^

TGF-*β*3	I+II	135	1.83 (0.69-4.88)	0.22	163	1.29 (0.69-2.43)	0.43
	III+IV	1220	1.14 (0.98-1.33)	0.095	1081	1.36 (1.18-1.56)	0.001^∗^

TGF-*β*4	I+II	135	2.96 (1.28-6.85)	0.0079^∗^	163	2.18 (1.22-3.87)	0.0068^∗^
	III+IV	1220	0.91 (0.78-1.07)	0.26	1081	1.17 (1.02-1.35)	0.029^∗^

^∗^ *P* < 0.05.

**Table 2:** Correlation of TGF-*β* subtypes with OS and PFS in ovarian cancer patients with different pathological grades of disease.

TGF-*β* subtypes	Pathological grade	Cases	OS	*P* value	Cases	PFS	*P* value
			HR (95% CI)			HR (95% CI)	
TGF-*β*1	I	56	0.61 (0.23-1.63)	0.32	37	0.12 (0.02-0.91)	0.0014^∗^
	II	324	1.41 (0.99-2.01)	0.057	256	0.85 (0.63-1.14)	0.27
	III	1015	1.10 (0.92-1.31)	0.3	837	0.83 (0.69-1.00)	0.056^∗^

TGF-*β*2	I	56	0.54 (0.21-1.41)	0.2	37	0.48 (0.12-1.58)	0.22
	II	324	1.40 (1.01-1.95)	0.045^∗^	256	1.33 (0.98-1.8)	0.069
	III	1015	1.30 (1.1-1.54)	0.0017^∗^	837	1.42 (1.20-1.68)	0.001^∗^

TGF-*β*3	I	56	2.31 (0.89-6.02)	0.076	37	1.90 (0.64-5.66)	0.24
	II	324	1.50 (1.09-2.07)	0.0012^∗^	256	1.68 (1.25-2.26)	0.0005^∗^
	III	1015	1.18 (1.00-1.39)	0.053	837	1.32 (1.12-1.57)	0.001^∗^

TGF-*β*4	I	56	0.66 (0.24-1.80)	0.42	37	7.86 (1.02-60.55)	0.019^∗^
	II	324	0.80 (0.59-.08)	0.14	256	0.80 (0.59-1.09)	0.15
	III	1015	1.16 (0.98-1.36)	0.0880	837	1.21 (1.02-1.43)	0.025^∗^

Notes: pathological grade I (well differentiated), pathological grade II (moderately differentiated), and pathological grade II (poorly differentiated). ^∗^*P* < 0.05.

**Table 3:** Correlation of TGF-*β* subtypes with OS and PFS in ovarian cancer patients with different TP53 mutation statuses.

TGF-*β* subtypes	TP53 mutation	Cases	OS	*P* value	PFS	*P* value
			HR (95% CI)		HR (95% CI)	
TGF-*β*1	Yes	506	1.16 (0.93-1.46)	0.19	0.79 (0.61-1.03)	0.085^∗^
	No	94	1.49 (0.86-2.58)	0.15	0.57 (0.32-1.03)	0.06

TGF-*β*2	Yes	506	0.86 (0.68-1.20)	0.22	1.25 (1.00-1.56)	0.049^∗^
	No	94	1.84 (0.86-3.91)	0.11	1.34 (0.76-2.37)	0.3

TGF-*β*3	Yes	506	1.38 (1.10-1.73)	0.0055^∗^	1.34 (1.07-1.67)	0.0099^∗^
	No	94	1.73 (0.99-3.02)	0.05	1.68 (0.95-2.97)	0.073

TGF-*β*4	Yes	506	1.28 (1.02-1.61)	0.035^∗^	1.39 (1.10-1.76)	0.0061^∗^
	No	94	1.50 (0.87-2.58)	0.14	1.31 (0.77-2.20)	0.32

^∗^ *P* < 0.05.

**Table 4:** Correlation of TGF-*β* subtypes with OS and PFS in ovarian cancer patients treated with different chemotherapy strategies.

TGF-*β* subtypes	Chemotherapy	Cases	OS	*P* value	Cases	PFS	*P* value
			HR (95% CI)			HR (95% CI)	
TGF-*β*1	Platin	1409	1.09 (0.95–1.26)	0.23	1259	1.08 (0.94–1.24)	0.27
	Taxol	793	0.86 (0.69–1.07)	0.18	715	0.85 (0.69–1.03)	0.094
	Platin+Taxol	776	0.88 (0.71–1.11)	0.28	698	0.86 (0.70–1.05)	0.13

TGF-*β*2	Platin	1409	1.20 (1.04–1.38)	0.0120^∗^	1259	1.46 (1.26–1.67)	0.001^∗^
	Taxol	793	1.27 (1.03–1.56)	0.025^∗^	715	1.43 (1.18–1.72)	0.0002^∗^
	Platin+Taxol	776	1.27 (1.03–1.57)	0.024^∗^	698	1.44 (1.20–1.74)	0.0001^∗^

TGF-*β*3	Platin	1409	1.20 (1.03–1.39)	0.016^∗^	1259	0.83 (0.72–0.96)	0.014^∗^
	Taxol	793	1.18 (0.97–1.43)	0.099	715	1.31 (1.10–1.55)	0.0024^∗^
	Platin+Taxol	776	1.19 (0.98–1.44)	0.085	698	1.31 (1.10–1.57)	0.0021^∗^

TGF-*β*4	Platin	1409	1.11 (0.95–1.29)	0.19	1259	0.85 (0.75–0.98)	0.02^∗^
	Taxol	793	1.13 (0.92–1.38)	0.24	715	1.15 (0.97–1.36)	0.12
	Platin+Taxol	776	1.16 (0.95–1.42)	0.16	698	1.17 (0.99–1.4)	0.07

^∗^ *P* < 0.05.

## Data Availability

The data used to support the findings of this study are included within the article.
